# Volunteer experiences and perceptions of the informed consent process: Lessons from two HIV clinical trials in Uganda

**DOI:** 10.1186/s12910-015-0073-1

**Published:** 2015-12-03

**Authors:** Agnes Ssali, Fiona Poland, Janet Seeley

**Affiliations:** Medical Research Council/Uganda Virus Research Institute (MRC/UVRI) Uganda Research Unit on AIDS, Kampala, Uganda; University of East Anglia, Norwich, UK; London School of Hygiene and Tropical Medicine, London, UK

**Keywords:** Informed consent, Clinical trials, Research volunteers

## Abstract

**Background:**

Informed consent as stipulated in regulatory human research guidelines requires that a volunteer is well-informed about what will happen to them in a trial. However researchers are faced with a challenge of how to ensure that a volunteer agreeing to take part in a clinical trial is truly informed. We conducted a qualitative study among volunteers taking part in two HIV clinical trials in Uganda to find out how they defined informed consent and their perceptions of the trial procedures, study information and interactions with the research team.

**Methods:**

Between January and December 2012, 23 volunteers who had been in the two trials for over 6 months, consented to be interviewed about their experience in the trial three times over a period of nine months. They also took part in focus group discussions. Themes informed by study research questions and emerging findings were used for content analysis.

**Results:**

Volunteers defined the informed consent process in terms of their individual welfare. Only two of the volunteers reported having referred during the trial to the participant information sheets given at the start of the trial. Volunteers remembered the information they had been given at the start of the trial on procedures that involved drawing blood and urine samples but not information about study design and randomisation. Volunteers said that they had understood the purpose of the trial. They said that signing a consent form showed that they had consented to take part in the trial but they also described it as being done to protect the researcher in case a volunteer later experienced side effects.

**Conclusion:**

Volunteers pay more attention during the consent process to procedures requiring biological tests than to study design issues. Trust built between volunteers and the research team could enhance the successful conduct of clinical trials by allowing for informal discussions to identify and review volunteers’ perceptions. These results point to the need for researchers to view informed consent as a process rather than an event.

## Background

Obtaining informed consent is required for all HIV clinical trials, in common with clinical trials worldwide, to ensure that volunteers who take part in the trial are well informed and are not coerced to join a trial [1–6]. Informed consent refers to rules that should guide health practitioners and researchers about how they interact with their patients or research subjects to enable truly voluntary participation [7, 8]. If they deviate from these rules, they are likely to face a penalty, particularly if such deviation affects the autonomy of a patient or research subject [9]. Judges at the Nuremberg tribunal enacted guidelines to provide standard laws on the conduct of human experimentation. These guidelines were needed because of the neglect by physicians and scientists of ethical conduct, including the principle of informed consent. These guidelines have since been subsequently revised and updated by medical associations and researchers [10].

**Table 1:** Comparison of the volunteer responses in the two case studies

Findings	Trial One responses	Trial Two responses
Perceptions of trial objectives	Investigating how the body would react to the study vaccines and whether it would cause harm	What would happen to their health if they stopped taking septrin
Side effects reported	Only 3 of 13 reported side effects which were: a swollen hand, dizzy feeling on first day of vaccination, heart palpitation but not related to high blood pressure	Only one volunteer reported that she became frequently sick since joining the trial
Reasons for joining the trial	Hope to protect from HIV	Hope to get improved quality of life, help others in future to take less drugs
Reasons for continuing in trial	Avoid catching HIV which information is reinforced by the research team at the scheduled follow up visits	It is a strategy to cope with HIV
Why a volunteer would drop out of the trial	Loss of interest, no time to attend follow-up visits, difficult with finding money to travel to research clinic	Failure to get money to travel for clinic visit, discouragement from other members in the community
^a^Who was involved in decision making	The female volunteers had to inform and sometimes seek permission from spouses. A few female volunteers made their own decisions. The male volunteers only make their own decisions and informed spouses but were not obliged to.	Both male and female volunteers made their own decisions and usually they would not share with their partners

^a^Trial One procedures required that a sexual partner was known and also tested for HIV; Trial Two volunteers were not obliged to bring their partners to the research clinic for HIV testing

Informed consent is fundamental to conducting research, as a principle in research ethics which enshrines the importance of respecting research subjects as autonomous and protecting their wellbeing [11–13]. Today the need to involve participants in research which is not necessarily therapeutic has increased the requirement to view informed consent as involving more than one interaction between the researcher and the research volunteer. In other words, to look at consent as more than a one-time event because the process involves a multiplicity of interactions between the key actors involved [14–17].

Informed consent, as an ethical principle used in conducting research with human subjects, has two main underlying ethics theories; the deontology theory and consequentialist theory. The deontology theory of ethics is concerned with protecting a person’s dignity and ability to self-regulate [17]. According to this theory a researcher must never use a volunteer to meet his/her (researcher’s) own ends unless the volunteer benefits from participating. The consequentialists on the other hand hold that ‘the rightness or wrongness of an act is dependent on the consequences of the act’ [14:19]. Our research, reported in this paper, incorporates more of the deontological theory,where the emphasis is volunteer participation in research where a person’s dignity is respected and he or she can make decisions during the course of the informed consent process.

Research experience has demonstrated that an on-going dialogue between researchers and research subjects, giving the latter time to reflect and ask questions and to consult family members or others that they trust, can enhance the informed consent process [18, 19]. Many studies, including those investigating participants’ comprehension of the information provided to them, have focused on understanding the issue of informed consent from different research volunteers and patients of what their perceptions were. Valley et al. [20], studied women who took part in a microbicide study in Mwanza and reported that the women were able to recall the key information about the study. However, whether their choice to participate in a trial was based on sound understanding of the benefits and risks of the trial or was a result of other factors such as trust, altruism and individual priorities was not clear. Smith et al., [21] and Behrendt et al., [22] have also assessed patients’ understanding of the informed consent process and note that patients’ understanding is less developed than what the physicians expected, especially about aspects of research design such as randomisation and procedures of randomised controlled trials. There has been other research that has looked at possible ways of sharing information between researchers, professional health staff and participants in ways that clarify what taking part in a study involves [23] or investigated the best tools for assessing participants’ comprehension of study information [3, 24, 25]. Mueller [26], an anthropologist who conducted an ethnographic study on the informed consent process and HIV clinical trials among people with HIV, emphasises the contingencies and uncertainties that volunteers and even researchers experience when conducting clinical trials. Other studies have examined the challenges that researchers in clinical trials may face in sharing information, such as differences in understanding or power between members of the public and researchers [27–31].

A systematic review of 21 studies on how informed consent was defined and measured in African research settings reported that comprehension is often poor among such study participants and this means that there is a need to develop a definition for informed consent which can be applied better in low literacy settings in Africa [32].

Researchers have argued that it is important to balance the international standards of good practice with responding to the situation in a given local context where research is being conducted [33]. The various studies conducted reflect the need to hear the voice of the research volunteers on their views and experiences when they take part in clinical trials.

The HIV-epidemic continues to pose a health threat to many people in Africa and elsewhere [34–36]. As a result, clinical trials to test treatments and prevention approaches continue to be required, for which volunteers need to be recruited and retained. Researchers have identified challenges they faced while conducting long-term clinical trials in Uganda which included high pregnancy rates among volunteers during trials coupled with high dropout rates, some due to pregnancy and others due to loss to follow-up [37–39].

Given that volunteers must understand what they are being asked to do in a trial and what the trial is for, it is important to study how effective informed consent processes may be in conveying this trial information, particularly in settings where education and literacy levels may be very varied, or the power differences between the researcher and researched are large. Literacy levels n Uganda were still relatively low (at 68.9 %) in 2011, the majority of the population was living below the poverty line (estimated at 84.9 %), the distribution of health facilities also varies between urban areas and rural areas. Although there are 10 referral hospitals in the country [40, 41]. The proportion of the population living within 5 kms of a health facility was 72 % in 2010, their access to health care facilities further limited by poor infrastructure, lack of medicines and other health supplies, shortage of human resources in the public sector, low salaries, and lack of accommodation at health facilities. In addition, the budget for medicines does not match the growing needs of the population [42].

In this paper we examine the process of obtaining informed consent reported by volunteers taking part in two HIV clinical trials in Uganda, considering volunteers’ views on the specific act of signing consent forms and the lessons these offer about the values, beliefs, gender differentials, power dynamics and decision-making patterns during the informed consent process.

The term 'informed consent' refers to giving all relevant information about what is involved in conducting and taking part in an HIV clinical trial, and ensuring that the person receiving this information has the capacity to make a decision and voluntarily consents to take part when they are satisfied that they have understood the information [19, 43, 44].

The 'informed consent process' in this paper refers to all elements of the process of providing informed consent, which will include mobilising the initial recruitment of volunteers, providing them with information about the study and what will happen to them if they take part, a review by an ethics committee of the informed consent documents and procedures and the signing of documents by a member of the research team with each research volunteer. It is important to note that this process also includes the various interactions that take place between the key actors over the course of a clinical trial and what their experiences were within the trial. Gaining informed consent is not only the moment of securing a signature; it requires an investment of the researcher and volunteer's time [23] including information sharing sessions long enough to ensure the volunteer understands what is to happen to them and what they are being asked to do.

Informed consent is fundamental to the ethical conduct of research [45], emphasising the importance of respecting the autonomy of research subjects and protecting their wellbeing [11–13] and must therefore be documented to be evidenced.

In the research presented in this paper we adopted the ‘process model’ of informed consent seen as a continuous exchange of information throughout the course of the health practitioner/researcher-patient relationship rather than an ‘event model’, which focuses on the short interaction involved in formal consent-giving so as to examine what happens over time in the context of these clinical trials [14]. The model sees decisions made in the health practitioner/researcher-patient interaction as continuous in all of the interactions that occur for the duration of the study [14, 15]. The process model allows for successive instructive exchanges of information between the health practitioner/researcher and the patient/volunteer offering multiple occasions for discussing the treatment or the research information. The patient/research volunteer and health practitioner/researcher will both ask questions and give answers during the informed consent process. This model assumes that the volunteer can make individual decisions during the interaction, opening for discussion the values, beliefs, gender differentials and power dynamics and decision making patterns of both parties. This gives greater transparency to decision-making and the roles of the researcher/physician and the volunteer/patient [46, 47].

The meaning and interpretation of the informed consent process for all actors needs to be understood in context to adequately capture the social and practical implications of the current rigorous formal process, which is guided by national and international research ethics guidelines [48–50].

## Methods

### Study design

This was a a qualitative study which involved a sub-sample of volunteers from two HIV clinical trials which are the cases in this paper [51, 52]. The volunteers for the qualitative study were purposively selected [53, 54]. The study explored the implementation of the informed consent process in two different HIV clinical trials in relation to how the informed consent process was constructed and interpreted and what was meaningful to each group of actors, including the trial volunteers [55, 56]. These two case studies (Trial One and Trial Two) were selected because they were both HIV clinical trials conducted by the same research organisation, going through similar ethical preparations before they were conducted. The two different trials facilitated a comparison since they were conducted in two different sites precluding any possible contamination between the volunteers. The analysis aimed to include participants’ perceptions and experiences on all aspects of the informed consent process and the factors that influence it in the longer term not just focusing on the single event of signing a consent form.

### Study area

The research was situated at two Medical Research Council/Uganda Virus Research Institute (MRC/UVRI) Uganda Research Unit on AIDS sites. Both case-study sites were in the central region of Uganda but drew on different types of communities. Most of the population of the Trial One site were involved in small-scale farming producing a few cash crops like maize and coffee. The Trial Two site was a semi-urban area near the shores of Lake Victoria where the population was mainly involved in fishing and selling fish, food businesses and running small kiosks. A few were farmers. Both study areas were affected by the HIV-epidemic.

The targeted volunteers in these trials differed in that in Trial One they were HIV-negative, and in Trial Two they were persons living with HIV but already prescribed Anti-Retroviral medications (ARVs) for at least six months.

### The two clinical trials studied

Trial One was a phase I double-blind placebo-controlled trial to evaluate safety and immunogenicity of the experimental vaccine drug F4co-adjuvanted with AS01B or AS01E and administered with the experimental vaccine Ad35- GRIN. The volunteers in the trial were adults aged 18–40 years. The follow up period of volunteers was 16 months.

Trial Two evaluated whether long-term primary and secondary prophylaxis with Cotrimoxazole (also known as Septrin) can be safely discontinued among Ugandan adults on antiretroviral therapy who have achieved sustained immune restoration (measured as a confirmed increase in CD4 count to 250 or more cells/mm). The trial was a phase IV double blind randomised placebo controlled trial. The volunteers were adults aged 18–59 years living with HIV. Follow up period was a minimum of 18 months and maximum of 36 months.

Both trials had screening and enrolling procedures. Blood and urine samples were being collected every three months from the volunteers.

### Volunteer recruitment and sampling

Twenty three volunteers were purposively selected from the participants in the two trials who attended for clinic visits when recruitment for this study began. Volunteers were selected to take part in the qualitative study if they were found at the clinic during the time the study was happening. The volunteers were accessed in two stages; first they were approached by research nurses working with the trials to briefly inform them about the qualitative research in which they could take part after they completed the trial procedures planned and if they were interested. Those volunteers who were interested in getting more information about the qualitative study were then sent to the qualitative researcher (the first author) to discuss the details of the qualitative study with them using the participant information sheets. If they agreed to take part, they then signed a consent form for the particular study. Recruitment of participants continued until the desired sample size was reached [57, 58]. Volunteers in the qualitative study were eligible if they had been in the clinical trial for at least six months, were willing to give additional time beyond the time required for their participation on the trial and had also signed a consent form for the qualitative study.

### Data collection and analysis

To gain an understanding of how the informed consent process was experienced and interpreted by the volunteers in the two HIV clinical trials the first author conducted semi-structured in-depth interviews and focus group discussions [54]. The interviews and focus groups were audio-recorded. The first author also carried out unstructured observation at the research clinics, observing the research activities and gaining insights on volunteer-research team interactions and among the volunteers themselves [59]. Data collection was carried out from January 2012 to Dec 2012. Data analysis was on-going and inductive throughout the data collection process, and facilitated in the final stages by using Nvivo 8 qualitative software to manage the coding. Codes were generated from the first six in-depth interviews and patterns drawn and themes created alongside the data collection process in consultation with the other authors [60, 61]. The findings were interpreted according to the themes identified in the data [62].

### Ethics, consent and permissions

This study was reviewed and approved by the Science and Ethics Committee (SEC) Uganda Virus Research Institute. The study was also approved by the International Research Ethics Committee of the School of International Development at the University of East Anglia,Norwich, United kingdom. It was cleared by Uganda National Council for Science and Technology (UNCST). Before contacting the respondents, the first author sought permission for including the two trials in the qualitative study from the site Principal Investigators. All interviewees gave their consent by signing a consent form before they participated in interviews and focus group discussions. The findings refer to the trials as either Trial One or Trial Two to which they were recruited but do not reveal names of volunteers.

## Results

### Characteristics of volunteers

The volunteers were aged between 19 and 50 years with the median age of 33 years. There were fourteen female and nine male volunteers. Two volunteers declined participation because they did not have time for the interviews and one was removed from the trial because of the results of diagnostic tests. These were not part of the twenty three included in the final study. All the volunteers knew their HIV sero status since it was a requirement for them for taking part in the HIV clinical trials. The volunteers were all working in their local area, some in formal employment like security guard but most were in informal jobs doing business, vehicle mechanics, hair and beauty salons, drivers and shopkeepers. Two female volunteers said they were not employed and one of the male volunteers was a university student. The trial nurses and mobiliser were the first trial staff contact and providers of the trial study information for the volunteers at the clinic. At every scheduled visit volunteers had to see a nurse before they moved on to other trial procedures, and it was at this point that volunteers were informed about the qualitative study. This paper does not present other actors’ perspectives.

### How volunteers understood study information

An important aspect of informed consent is the discussion of the trial information between the research team and the volunteers. The discussion of study information was meant to ensure that the researcher and volunteer understood what was going to happen during their interaction and what procedures, risks and benefits a volunteer would go through. As Appelbaum and colleagues [14] say, ‘The duty of disclosure or duty to inform is the truly distinguishing and innovative aspect of the informed consent doctrine’[14, 57]. The volunteer perceptions on the trial information disclosed to them were important in pointing to the importance of disclosure of trial information.

The research teams and scientists proved especially keen to provide the volunteers with information about the trial and to ensure that they understood the research aims [63]. The volunteers on the other hand proved to be more interested in the practical procedures such as taking off their blood and urine samples and the state of their health whether they were still HIV negative or had an increased number of antibodies for those who were HIV positive.

Most of the thirteen Trial One volunteers did not see the vaccine given to them in that trial as an intervention to prevent HIV but to investigate how their bodies would react to it including whether it caused them harm.

*R4: They told us … that the vaccine we are being given, they said some will get the real drug and others will get a placebo; anyway the vaccine is not to prevent HIV but they want to see how our bodies will react to the drug.*(FGD,Trial One)

One female volunteer reported that ‘*they are just testing this drug on us to see if it cures AIDS, but it does not prevent it’*, adding that she needed to protect herself from acquiring HIV. This reflects the need for continuos dialogue between the volunteers and the research team to deal with misinterpretation of study information.

All Trial One volunteers knew that the trial involved randomization, while nonetheless they all hoped they were on the active and not the placebo arm of the trial. This seems to reflect their belief about the trial that once one is given a drug it must be useful to them while also sometimes doubting the completeness of the information the researchers gave them.

*R7: That is the truth because the researchers* (abasawo), *they hid something from us …Now among the nine of us, we cannot know whether five got a placebo or four got the right vaccine. We are looking at each other because they’ve kept that to themselves [all laugh], but when they look at us, I think they say ‘Because of what I see, I think what we gave him works’, because for them, they know.*

*R6: Even the researchers do not know.*

*R7: But for them they know the bottle types where they got the drug to inject us. [All laugh]*(FGD,Trial One)

When asked what the objectives of the trial were, Trial Two volunteers commonly suggested that it was to test what would happen to them if they stopped taking Cotrimoxazole (Septrin):

*They are researching so that they can see whether we can stop taking Septrin or we need to continue taking it.* (Female volunteer aged 40, Trial Two)

Despite all ten volunteer interviewees in Trial Two reporting that they were involved in research on Septrin and the effects of stopping taking it, only four of them could directly articulate that there was randomization in the trial so that some volunteers were on a placebo and others on the active drug. Two Trial Two volunteers reported in the focus group that the trial was ‘… *trying to find out the effects of stopping taking Septrin but continuing with ARVs*’. The remaining eight volunteers did not clarify what randomization meant for them personally. One discussant said:

*We hoped that if we left that strong Septrin and remained on the ARVs and the drug they are giving us we would survive. They also told us that if it doesn’t work, and you get weak after leaving Septrin and you’re getting funny effects, you can get back on the old Septrin. So we got hopeful and said ‘Why don’t we join – we’ll probably get a cure‘.* (FGD Trial Two)

This quote above raises several issues about volunteer comprehension of study information. First, the volunteer mentions that they were told they would be put back on Septrin if they became sick; this was not written in the trial information sheet however it was part of practice according to the trial protocol. She also mentioned that they had been taken off the ‘strong Septrin’, implying that they may be on some form of drug which is not so strong, and hopes that by joining the trial that they could be cured. The volunteers interviewed  in this qualitative study had all been in the trial for at least six months to a year.

Trial Two volunteers had more issues with the kind of information that they received about the trial than those in Trial One. While they all first mentioned that the trial was about Septrin, their discussion of randomization and any expected trial outcomes were mixed up with rumours from other volunteers. It was not ultimately easy to tease out which facts about the trial were clear to them and which ideas about the trial might have developed, perhaps for example from their own discussion of the trial, some time after the initial information had been given to them.

### Decision-making by the volunteers

Most Trial Two volunteers decided to join a trial in the hope that the research would eventually lead to a reduction in the number of pills they had to take, while Trial One volunteers hoped that it would protect them from HIV infection. All volunteers described their participation in the trial as voluntary. However, they also described seeking advice from someone they trusted such as a parent, counsellor or respected community leader before agreeing to take part.

Although the volunteers had made the decision to join a trial, some reported later difficulty in keeping to their decision and continuing in the trial. Trial One volunteers reported being discussed in the community, where it was believed that they had joined a clinical study because they were infected with HIV. There was stigma attached to attending the research clinic in one of the study areas because the community believed that since the MRC conducts HIV and AIDS-related research all volunteers must be HIV positive. Thus communities still evoke stigma despite having lived with HIV and AIDS in Uganda for over two decades [36]. The other difficulty faced by Trial One volunteers both before and after deciding to participate were fears raised by the rumours about what others said would happen to them:

*Before making the decision we talked about this research with our friends … and got different feedback, such as ‘They are going to vaccinate you and you don’t know what will happen to you in ten or twenty years’: ‘You might grow a lot of hair like monkeys’ [all laugh] or ‘You will die a slow death’ … It was hard, and we actually had to think hard before volunteering.* (Volunteer FGD, Trial One)

The volunteers in Trial One were anxious at the start of the trial, several wondering why the research team were not taking part in it themselves. Volunteers in the focus group reported their need for reassurance from the researchers that the potential harm discussed in the community would not happen to them. Both trials’ research teams maintained continuous dialogue with the volunteers to help allay such anxieties.

Some volunteers’ decisions, particularly about Trial One, were delayed because of having to inform their spouses, as encouraged by the researchers, and some spouses did not support their joining the research and even stigmatized them for it:

*My husband would ask me how things had been [at the clinic] and he would tell me why [the researchers] do not try those vaccines on animals … sometimes I got the signs that they had told us about, some feverishness and feeling cold … Every time I felt a bit sick he would tell me that I would die in a few days.* (Female volunteer, Trial One FGD)

While some volunteers faced challenges when deciding whether to join a trial, others did not, either because they had support from someone such as a parent or because they were independent in making their decisions, as was more common among the men. Gender norms in this community required that married women made important decisions after informing and sometimes after getting permission from their spouse; this was an issue reported more by Trial One volunteers because enrolling in the trial required their spouses to be tested for HIV. Both male and female volunteers in Trial Two were more independent in their decision to join the trial because they felt that it was up to them to help to find a solution to HIV-infection, which was already a challenge in their lives, and with no trial requirement of HIV testing for their partners.

Deciding to remain in the trial meant that some volunteers had to continuously make decisions, such as whether to refrain from sexual relationships:

*You may admire someone and want to have sex with them, but because you are taking part in research you decide to wait until you are done with the research. (Male volunteer aged 24, Trial One)*

Thus individual decision-making patterns differed for each volunteer according to their social context. The rumours of their being infected with HIV in the trials made it hard for some of those seeking good health to decide to take part.

#### Who influences the decision to participate in research?

Female volunteers confided mainly in their parents before deciding to join the trial. However, most of the Trial One male volunteers did not consult anyone, and the few who did asked the advice of friends or elder siblings. Some married male volunteers informed their partners of trial details including expected duration of the trial to avoid conflict especially because after the initial enrolment they still had to come to the clinic for follow up visits:

*… this depends on you as an individual, but you need to inform your partner because if you do not tell them [what happens at the clinic] and yet I usually go home with leaflets with the MRC information … It might give her the idea that you’re probably HIV positive and you haven’t told her.* (Male volunteer aged 31, Trial One)

This comment reflects the type of community stigma that is attached to volunteering for research. In this society, men are not obliged to consult anyone before making their decisions but, as seen here, the issue of HIV and AIDS may raise questions in some relationships.

Many Trial Two volunteers made their decision to join the trial alone, because they are living with HIV infection:

*I did not seek [my husband’s] advice before I joined the trial. When l heard what they told me about the research, I decided on my own to join. My husband learnt about it later.* (Female volunteer aged 25, Trial Two)

One female volunteer did not disclose her trial participation to anyone, including her sexual partner at the time:

*I made my own decision. Although I live with a man he does not know I’m taking part in a trial.* (Female volunteer aged 40, Trial Two)

The volunteers’ privacy was important to them even though the informed consent process might involve discussion between family members, the volunteer and other actors in the research process.

As many volunteers often referred to consulting important others in their lives before making their final decision to join a trial, if they were to keep in the trial for the whole duration of the research, researchers should take the time to find out who these important others in a volunteer’s social network who might impact on trial participation [64].

### Roles in the informed consent process

Volunteers were asked to describe their roles in the informed consent process to find out how they value their own and other actors’ contribution. Volunteers often demonstrated a precise awareness of this:

*My role is important because if we don’t enrol then who will they conduct the research on? Will they do it on animals? So that all shows that the research team has to handle us well.* (Female volunteer aged 26, Trial Two).

Another volunteer explained her involvement as a way to responsibly encourage other people to join research:

*My main responsibility is to avoid getting HIV and to discuss information that l have with other people … after this experience when I meet people and there is research being done, I encourage them to join.* (Female volunteer aged 30, Trial One)

The volunteers said that their own role was to keep their appointments, and once at the research clinic, to follow what was planned for that visit. A volunteer described a typical follow- up visit day:

*When I arrive I give in my card, they write my name and then I wait for about ten minutes. Then I go to see the doctor; from there I come back and they take my blood, then they reimburse my transport costs, then I get the drugs and I leave.* (Female volunteer aged 50, Trial Two)

All the other respondents including research teams, ethics committee members and community advisors represented the volunteer as central to the informed consent process.

### Reasons for participating in an HIV clinical trial

The reasons volunteers gave for joining the clinical trial were related to their experiences with HIV and AIDS. The effects of HIV or present HIV-infected state led them to participate. The volunteers in both studies expected that a solution to the HIV problem would be found in the future. Trial One volunteers hoped that they would be protected from acquiring HIV infection while the Trial Two volunteers expected to lead a better quality of life as a result of the trials. These two issues point to forms of therapeutic misconception in each case with Trial One volunteers hoping participation would lead to protection from acquiring HIV and for Trial Two volunteers hoping to gain improved quality of life.

There were also marked differences between the two groups of volunteers’ reasons for continuing to take part in trials. Whereas Trial One volunteers were concerned with avoiding catching HIV, Trial Two volunteers sought strategies for helping them cope with their HIV-positive status, in line with their cultural obligations, beliefs and expectations of the trial with a few reporting an additional reason as being to help others benefit from the research finding that fewer people would be taking too many drugs.

All Trial One volunteers reported their main reason for continuing trial participation was their desire to protect themselves from catching HIV because of the study information on consistent condom use that they were reminded about during the clinic follow up visits. When asked how being in a research trial could help with this, the majority said that the research team’s HIV-prevention messages such as ‘consistent use of condoms for every sexual act’ were constant reminders that the intervention vaccine did not protect them from acquiring HIV.

The typical first response of Trial Two volunteers about why they stayed in the clinical trial was their expectation of medical advances from the trial:

*I expect we will eventually get a drug that will cure us, and I will get cured myself.* (Female volunteer aged 40, Trial Two)

Two male volunteers in Trial Two hoped to each have a son, in the future. One of these men was happy to report that his pregnant wife was still HIV-negative:

*I’m still lucky; my wife is HIV-negative … now she is pregnant, about to have a second child. And if God helps me and it is a boy then I will be one of the luckiest.* (Male volunteer aged 39, Trial Two)

The other male volunteer also hoped one day to have another child:

*I have been hoping that in the near future I will be fine and be able to get a woman and have a child, probably an heir, because currently I only have one child.* (Male volunteer aged 50, Trial Two)

While this volunteer already had a wife and one child, after many years without another child the beliefs and values of this society underpinned his desire to acquire another wife and probably another child [65–67] . These two examples show the type and degree of emotional and psychological stress that people living with HIV may feel while trying to cope with HIV infection.

### Feelings about the time required for the trial procedures

Taking part in the trial procedures may present the volunteers with many difficulties. Many in Trial One reported how challenging they found the length of time they needed to spend at the research clinics.

This was not the case for most Trial Two volunteers, some of whom favourably compared their shorter waits to collecting their ARVs in the research clinics than from service institutions.

A Trial Two volunteer explained the importance of the shorter time spent at the research clinic:*There is such a big difference between institution D [a service institution] and this trial clinic. In D you may wait from morning to 2 pm for your turn to be seen by the counsellor or clinician – at least here they work fast and then you can go back and do your work .* (Female volunteer aged 26, Trial Two)

Trial Two volunteers reported delays at the pharmacy when they attended the clinic. Observing the pharmacy, it was apparent that one research team member usually worked alone providing refills and dealing with prescriptions for all research volunteers. This was a slow process and a source of some discomfort for some volunteers.

Trial One volunteers repeatedly related the acute problem of the longer time spent at the clinic, to their job commitments; identifying what they gave up to attend, mainly in monetary terms. A focus group discussion quote highlights such concerns:

*I think there are too few doctors. Although we agreed to be volunteers we have other things we want to go and do. I think the issue of time can actually cause people to drop out, even if they volunteered willingly.* (FGD Trial One)

Despite volunteers’ complaints about lost time, the research team had to take the volunteers through the planned procedures on each scheduled clinic visit. This could indirectly affect the way research procedures are managed by the research team, because they had to work very fast in response to the volunteers’ demands.

The Trial One volunteers in this study were not often sick, and it was not easy to handle their time because even on arriving for their interviews for this study they always began by announcing how little time they had, which reflected their concerns about time taken up by the trial. A Trial One volunteer suggested that the research team should separate the volunteers who require health care from those invited for scheduled research visits to minimise time spent at the clinic.

Since time was seen to be critical to the volunteers in Trial One, the research team members were interviewed about their views on the time taken by the research procedures. All team and SEC members reported having enough time to share trial information at the start of a trial as very important when conducting a trial to enable volunteers to ask questions and have them answered by the research team, to help them understand the study they are involved in and its rationale.

The research team and SEC generally reported that spending time on explanatory procedures, especially at the start of a trial, is essential*.* This view was however challenged by my finding that procedures such as blood and urine sample collection were reported spontaneously by volunteers while other procedures were less significant to them, so spending much time on explaining such procedures was not welcomed by them.

The volunteers offered some suggestions for reducing time spent at the clinic, including dividing the research team into two groups, one to take care of sick volunteers and the other to handle research volunteers’ scheduled appointments.

### Reasons why volunteers drop out of trials

Volunteers were interviewed about reasons why some dropped out of research trials. Their reasons included losing interest in the trial, not having time for follow-up visits, and difficulties finding money for transport to the clinic. While the research team refunded transport costs after a scheduled visit, some volunteers could not find enough money to attend the clinic and ended up dropping out of the trial. See Table 1 below  for the summary comparison of responses from the trial volunteers in the two trials.

### Volunteer experiences during the informed consent process

When describing the study procedures in the informed consent process, in their first interviews with the first author, the volunteers detailed what happened to them from first walking into the research clinic, and the information the research team provided. Explaining what procedures volunteers found important during the three monthly follow–up clinic visits, most commented that nothing about the trial procedures they underwent had changed. It may have been the case that nothing had changed, these responses may partly have been because the second time they met the interviewer (first author), she was no longer a stranger to the research so they expected her to know what was going on or because the procedures had become ‘usual’ to them and therefore did not require further reporting.

When interviewed about specific procedures the volunteers all mentioned that research teams collected blood and urine samples from them, easily describing the different procedures they went through and knew what to expect at their scheduled visits. This reflects the trust they had in the research team. Volunteers described such procedures subsequently as “usual procedures” during their second and third interviews, while always reporting that they had given a blood and urine sample during the follow up clinic visits:

*They were the usual [procedures]; of course when I went they took my urine sample and a blood sample.* (Female volunteer aged 25, Trial One)

Another female reported that she had been reprimanded by research team members because she had been sick and had not come to the clinic for treatment, as required of her as a volunteer:

*I told them what I had gone through and that I had been sick but had not reported it and they rebuked me because they had told us to come when we are sick. They took a blood sample and then they gave me more Septrin.* (Female volunteer aged 40, Trial Two)

The regular clinical procedures reported here were expected by the volunteers. However, as seen in the second quote above volunteers may not always adhere to everything required of them as specified in the information they had discussed earlier with the research team.

### Significance of signing and thumb printing the consent forms

Volunteers were asked what significance they gave to signing the consent forms and how they would gauge whether someone had understood the trial information that the research team provided. All volunteers reported that it was very important that they sign or thumbprint the consent forms and that informed consent must be documented; most said that signing the consent forms showed a volunteer agreed to participate in a trial without coercion.

Most volunteers discussed signing or thumb printing the consent forms matter-of-factly, as a usual procedure, perhaps indicating that they were used to the way research studies are run. The volunteers reported signing a consent form as part of ensuring their formal agreement to join a study. The volunteers in these two trials stated that there was no alternative to signing or thumb printing the consent forms in research:

*Even if I’ve agreed to participate, you cannot know [that I have agreed to take part in the trial] until someone puts it in writing. You know things that you just talk about are not easy to accept unless you have put something in place.* (Female volunteer aged 30, Trial Two)

The volunteers reported that signing or thumb printing a document was not a challenge for them, and saw refusal to sign the consent forms as indicating a potential volunteer’s lack of interest in a study.

Volunteers able to write were asked what they thought about the possibility of being asked to provide a thumbprint on the consent forms, a required practice in research before enrolling volunteers unable to read or write. The volunteers felt it was not right to ask someone who could write to sign with a thumbprint. It was prestigious for a volunteer to be able to sign the consent forms, but there was stigma attached to thumb printing.

A male volunteer mentioned that thumbprints may be used in banks and elsewhere, but asking a research volunteer to thumbprint the consent forms when able to write their name was not acceptable. To some volunteers however, it did not matter whether they signed or thumb printed the form.

Signing the consent forms was a research requirement which the volunteers accepted without question. The main reason most volunteers gave for the necessity of signing was to show that they had not been coerced; they did not mention anything related to study content such as the trial’s objectives and procedures as presented by research teams at the information sessions.

Most volunteers felt that it did not matter whether one signed or thumb printed; what was important was being able to understand the information given by the research team:

*We need to know that both a person who has understood and one who has not understood can sign.* (Female volunteer aged 31, Trial One)

### Volunteers’ suggestions for the informed consent process

The volunteers made several suggestions about improving activities that took place during the research, all of which related to the informed consent process including: gauging their understanding after being in a research trial for a while, as testing for comprehension of study information soon after providing it might only have shown that they could recall it superficially:

*Questions should not be asked immediately, because sometimes we are asked questions about what we have just been told; the brain is still recalling what you read to me some ten minutes ago. (*Male volunteer aged 27, Trial One)

The volunteers also suggested that all study information should be simple and clear:

*When we are given information it does not tell us definitely what will happen to the baby [if a volunteer gets pregnant] – they always say the baby ‘might have a problem’. Even the people who give the information are not definite.* (Male volunteer aged 27, Trial One)

The consent document for one trial stated; ‘We do not know what effect the vaccine would have on an unborn child if given to a pregnant woman. If you become pregnant during the study, you will not get any more vaccinations’ (ICD). While the research team may have easily understood this sentence, the volunteer’s concern was that it left room for the volunteer to decide whether to get pregnant or not.

They also suggested that the research teams should hold more than a single information session to ensure that volunteers understand the trial objectives.

Volunteers suggested that the research teams should devise an adult literacy programme to support volunteers who cannot write their names, during long-term trials so that volunteers unable to write their names at the start of a trial would be able to do so by the end, as a useful benefit for volunteers. This suggestion highlights the community stigma attached to volunteers unable to sign his/her name on the consent forms. The suggestion persisted through later interviews and the focus group discussions despite all volunteers having noted that understanding the study information has nothing to do with ability to sign consent forms.

Other suggestions to improve the informed consent process were personal, such as requesting for more money for transport to the clinic because travel costs fluctuate. A few female volunteers suggested that research should not include females aged 25 years or younger, who still needed to have children and might not wait for a long time of the trial before they became pregnant. Young wives were expected by their husbands to get pregnant when they have been married. Another suggestion related to reproductive health was that clinical trials should only enrol non-sexually active women. Both suggestions relate to the sociocultural norms in this community; once married, a woman is expected to produce children as soon as possible [67]. A male volunteer suggested that female volunteers should be given hormonal family planning methods such as contraceptive injections to avoid pregnancy during a trial.

A focus group discussion with the volunteers suggested to the importance of informing them about all research activities taking place at the research clinic. For example, they suggested that the research teams should explain when there were multiple research projects going on simultaneously to help volunteers understand that sometimes they would have to wait for a while before being called in to see research team members who were carrying out the scheduled trial procedures.

## Discussion

The informed consent process in this study focussed on the volunteer perceptions and experiences of the informed consent process, which underlined that the interactions and communications between volunteers and research team vitally supported understandings of the consent process similar to what was found in Kenya [64]. Here we discuss volunteer understanding of study information, decision making patterns, their roles and experiences during the informed consent process and their views on signing and thumb printing consent forms.

While the researchers may spend much time presenting the volunteers with the trial overview information such as study design, trial key messages, planned interviews and clinical examinations to be conducted, this study has found that at the start of a trial volunteers may not consider all of this information important. The volunteers were keen to report the procedures that involved giving blood and urine samples at clinic visits. Volunteers reported these more readily than general information provided about trial objectives. It may be more appropriate to discuss such information piecemeal and in stages as the volunteer progresses through the trial.

The volunteers again alluded particularly to respect-related issues when discussing their comprehension of the study information. They suggested that a non-literate individual was able to understand this information as well as a literate individual when it was discussed in their own language. Therefore whether a volunteer signed their name or they were only able to make a mark using their thumbprint on the consent forms, they did not see this as reflecting their ability or failure to comprehend study information.

Research ethics guidelines emphasize that non-literate volunteers must have literate witnesses present when they receive the study information and when signing the form consenting to take part in the research [2, 68, 69]. Some volunteers saw the signed consent forms as legal documents binding the researcher and the volunteer in a research relationship for the trial’s duration. If volunteers view the consent forms as legal documents, they may not ask questions pertaining to their participation in research since they would assume that they are bound by signing the document. This may imply inequality between research volunteer and researcher which may not foster continued collaboration. Only two volunteers reported having referred back to their signed consent forms. When a few of the volunteers reported in this study referring to the trial information sheet, it underpins the importance of having an on-going informed consent process as the researcher and volunteer interact in order to ensure true informed consent [49].

Decisions on whether to join a trial and continue participation can be influenced by the individual volunteer’s values and beliefs, their trust in the research team, the power dynamics during the interactions between the volunteer, relatives and research team. A study by Kingori reported that many participants made the decision to join research even before they were given study details [70]. Social cultural factors in their community also played a role in how they made decisions to join or continue in a trial, gender-related issues were prominent, especially for women who in most relationships made reproductive health decisions with permission of or in consultation with their spouses [66, 71].

The volunteers based their decision to take part in the trials on their trust that the study information provided by the research team and the trial procedures they would undergo would not cause them harm. This shows the trust built between the volunteer and the research team while interacting with each other as a factor that may lead to the volunteer’s continued participation in a clinical trial which trust has also been noted in neighbouring Kenya [20, 64].

Volunteers’ main reason for taking part in the clinical trials related to past and present experiences with HIV and AIDS, some volunteers having experienced deaths of close family members and friends and some were living with HIV. Some volunteers gave altruism as one of their reasons for taking part in research, yet all the volunteers named routine health checks as the main benefit from being involved in research.

In this study, the standardised national research ethics guidelines were particularly emphasised as important by the ethics committee and the senior scientists conducting the trials, who had to ensure that the procedures research volunteers went through were aligned to what the guidelines stipulate. We found that the nature of the trial and the research setting may contribute to the recruitment and retention outcome from the informed consent process which result is similar to the findings of Molyneux and colleagues, for parents who consented to their children joining a vaccine trial [48].

We found in our research that in the prevention trial where the volunteers were not ill, they could easily decline to join the research if they found that they were to spend a lot of time for which they had other priorities. Those volunteers in a treatment trial, were unlikely to press hard to limit the time they needed to spend at the clinic because they believed that good health was dependent on taking drugs and obtaining good health care from such a research clinic.

The volunteers’ discussion of informed consent had nothing to do with the technical expectations laid down in the human research ethics guidelines. They showed little awareness of principles of documented research, yet they had been provided with specific information about the trials that they had joined, and with copies of the consent forms they had signed when being screened and enrolled. While it could be argued that this was a procedure enacted by the research team to accord volunteers respect by asking for their consent individually, it is also a research ethics requirement while conducting research [2, 6]. Beyond the signed consent forms by the volunteer and the research team member, there was no documentation to show how the on-going interactions between the research team and the volunteers were managed to protect the volunteer.

Volunteers may continue to participate in research because of their personal aspirations which may not necessarily relate to the objectives of a trial. Volunteers’ expectations related to the individual; how they would be protected from foreseeable risks relating to the trial and might benefit from taking part in the trial [72]. A male volunteer who had felt more constrained from having sex with strangers noted that taking part in the research enforced positive behaviour such as consistent condom use with every sexual partner and saw this as a benefit for him.

Although the participant information sheet indicated that the volunteer was free to take part in a trial and to withdraw at any time, it also spelt out how trial protocol-based procedures needed to be followed by the volunteer, to do what was required of them by the research team after agreeing to take part. The findings show that volunteers may not give equal attention to all issues the research team emphasized, such as reporting to the clinic whenever they were unwell when other options such as getting treatment from a nearby drug shop might have been available to them. All such factors meant that volunteers might not always adhere to the research team’s expectations.

The research team and volunteers did not always, therefore, attach the same importance to aspects of the informed consent process. This shows not only that the volunteer is dependent on the research team but also that the research team is dependent on the volunteers’ input to the informed consent process, in terms of consenting to adhere to the trial procedures.

The volunteers’ opinions about decision-making and consenting to take part in clinical trials demonstrated their active and sometimes changing involvement in the informed consent process and their ability to reflect and comment on what they observed during the research process.

Volunteers’ suggestions for the informed consent process showed their interest in what would happen to them during research and also that significant others were critical in their decision to join a trial [64]. They also highlighted that study information needed to be presented clearly to overcome misinterpretation of key study messages and procedures by the volunteers. They also emphasised that volunteer comprehension of this information was not simply and only linked to literacy,but may be affected by other factors such as participants’ ill health or wellbeing [73].

### Study limitations

The main limitation was that the purposive selection of volunteers may have led to some bias in who was invited to join the qualitative trial. The study was conducted at the clinic setting which may have influenced the volunteers’ freedom to respond to the research questions since clinical procedures were being conducted at the clinic. The main researcher could not observe volunteers- clinic team interactions within the clinic room which meant that she had to rely on reported information and observations outside the clinic rooms.

## Conclusion

Although the regulatory guidelines provide a description of what the informed consent process should involve for individual participation, in practice the beliefs, values, trust and power dynamics during clinical trial implementation were seen to influence the consent process in which the volunteers and the research team interacted with each other and with their wider social networks. The suggestions by volunteers for improving clarity and their comprehension of trial information during the informed consent process require responses from the research community particularly as these highlighted how scientific terms used may not be well-understood after translation into lay terms. Volunteers’ comprehension of key information in trials should not be assessed immediately after information-giving because what they convey may be literal recall rather than necessarily a full understanding.

The informed consent process is not only about formal enactment of regulations as isolated events, but also depends on the continuing interactions between the research team, the research volunteers and their communities throughout the trial.
